# Associations between echocardiographic parameters and aerobic performance in young athletes

**DOI:** 10.7717/peerj.21263

**Published:** 2026-07-14

**Authors:** Alkame Akgümüş, Ahmet Kurtoğlu, Ömer Özer, Ali Duygu, Bekir Çar, Ertuğrul Kurtoğlu, Kamil Uzgur, Fahri Er, Jarosław Muracki, Safaa M. Elkholi

**Affiliations:** 1 Department of Cardiology, Medical Faculty, Bandirma Onyedi Eylul University, Balıkesir, Turkey; 2 Department of Coaching Education, Faculty of Sport Science, Bandirma Onyedi Eylul University, Balıkesir, Turkey; 3 Department of Physical Education and Sport Teaching, Faculty of Sport Science, Bandirma Onyedi Eylul University, Balıkesir, Turkey; 4 Department of Cardiology, Medical Faculty, Malatya Turgut Özal University, Malatya, Turkey; 5 Institute of Physical Culture Sciences, Department of Physical Culture and Health, University of Szczecin, Szczecin, Poland; 6 Department of Rehabilitation Sciences, College of Health and Rehabilitation Sciences, Princess Nourah bint Abdulrahman University, Riyadh, Saudi Arabia

**Keywords:** Echocardiography, Aerobic performance, Cardiac morphology, Cardiac function

## Abstract

**Background and Objectives:**

Cardiac morphology can be shaped according to chronic and acute exercise and provides important findings regarding cardiac response to different types of performance. However, cardiac remodelling is a multidimensional process involving intertwined structural, functional, and haemodynamic components, making it difficult to explain with a single criterion and requiring a comprehensive assessment. The aim of this study is to investigate the effects of echocardiographic (ECHO) parameters on 30–15 interval fitness test (IFT) performance.

**Materials and Methods:**

Twenty-three active athletes participated in the study. All ECHO measurements were performed by a specialist cardiologist using standard clinical methods, and the IFT was administered to participants after the measurements. In the analysis, ECHO variables representing the structural characteristics of the left ventricle, systolic-diastolic function, and aortic/atrial measurements were evaluated, with priority given to parameters indexed according to body surface area (BSA). Relationships between IFT performance and ECHO parameters were examined by establishing parsimonious linear regression models appropriate for small sample sizes; models were evaluated using Akaike Information Criterion (AICc)-based model comparison and model averaging, results were checked for robustness using leave-one-out cross-validation (LOOCV), and the SHapley Additive exPlanations (SHAP) approach, consistent with the model, was used for interpretability.

**Results:**

In the AICc-based model comparison, the parsimonious model with the highest support consisted of the body composition (BMI) + posterior wall (PW)_I + aortic strain (AORT_STR) components and explained approximately 46% of the variance in IFT performance (mean adj. *R*^2^ = 0.461; LOOCV root mean squared error (RMSE) = 1.811; mean ΔAICc = 0; mean Akaike weight = 0.0476). Variable importance analyses in the best model set revealed that the variables most consistently associated with IFT were BMI (Σ*w* = 0.46; weighted mean—SHAP— = 0.154; *β* =  − 0.068) and posterior wall thickness indexed by BSA (PW_I) (Σ*w* = 0.35; weighted mean—SHAP— = 0.211; *β* = 0.348). This was followed by IVS_I (Σ*w* = 0.191; weighted mean—SHAP— = 0.072; *β* = 0.124), A wave (Σ*w* = 0.177; weighted mean—SHAP— = 0.080; *β* = 0.849) and AORT_STR (Σ*w* = 0.166; weighted mean—SHAP— = 0.077; *β* = 0.013) followed. Overall, the distribution of model weights across multiple models indicated significant model uncertainty, while BMI and indexed structural measures provided the strongest and most consistent signal in relation to IFT.

**Conclusions:**

In this context, it has been concluded that body composition is also a determining factor in the relationship between cardiac morphology and IFT performance. Large prospective studies are important for determining this complex relationship.

## Introduction

Aerobic performance provides significant outcomes in enabling individuals to sustain a particular exercise or activity for longer periods (Manresa-Rocamora et al., 2021), in replenishing depleted energy stores after exercise (Tomlin & Wenger, 2001), and in accelerating physical recovery following high-intensity exercise (Jarstad & Mamen, 2019). Although sprinting, acute changes of direction, or vertical jumping are anaerobic performance indicators, athletes also need a high aerobic capacity to sustain this performance for long periods (Harrison et al., 2015). Therefore, in order to achieve high performance levels in athletes, internal, external and environmental factors affecting aerobic performance must be well optimized. Therefore, it is important to reliably assess aerobic capacity under field conditions and to consider performance-related physiological determinants together.

The 30–15 interval fitness test (IFT) is a field test frequently used to estimate maximal aerobic speed and maximal oxygen consumption utilised by athletes (Buchheit, 2008; Scott et al., 2017a; Scott et al., 2017b). The physiological basis of aerobic performance largely depends on cardiovascular capacity (Joyner & Dominelli, 2021). Echocardiographic (ECHO) findings are crucial in determining these characteristics of the heart. These findings are important in assessing the cardiac response at rest and during exercise by determining both the structural reserves and functions of the heart (Kurtoğlu et al., 2024). They also provide important outputs to coaches and clinicians in predicting the anaerobic and aerobic performance profile of athletes.

Echocardiographic data gives insight into heart adaptation to training in a manner that might correlate with aerobic exercise performance in field testing, although connections between heart structure and function and exercise performance are far from direct: several aspects could play a role simultaneously, with their combined effect possibly varying between subjects even in populations with a similar level of training (Rajiah et al., 2025). In the context of sports cardiology, with small populations to work with, it is easy to find oneself misled by correlations without paying attention to the combined context, which is why explainable modelling approaches might find their use, without any claim of actual causality, to indicate which variables tend to feature as significant together (Collins et al., 2024). In such situations, the aim is not to claim causality, but to transparently present the co-varying determinants. Due to this multi-dimensional structure, interpreting the relationship between ECHO parameters and performance by looking at individual variables alone is often insufficient; there is a need to explain the contribution of variables to the model collectively and transparently. At this point, the SHapley Additive exPlanations (SHAP) method separates the contribution of each parameter to the model output, revealing which variables are more effective and significantly contributing to the understanding of the model’s key determinants (Mangalathu, Hwang & Jeon, 2020). In previous studies, the SHAP method has been used to determine the effect of biomarkers on cardiovascular mortality (Wang et al., 2021), to classify risk in heart failure (Luo et al., 2024), to predict exercise-related physiological responses (Ali et al., 2024), and to predict other diseases (Haskul, Kaya & Kurtoğlu, 2025). This also demonstrates that SHAP analysis can identify complex relationships between parameters in complex datasets with high accuracy.

In this study, a parsimonious and clinically interpretable modelling strategy was adopted to reduce the risk of overfitting under small sample conditions. Therefore, this study aimed to investigate the relationships between ECHO parameters and 30–15 IFT performance using an inference-focused and clinically interpretable modelling framework. Given the multidimensional nature of cardiac structure and function, a series of conservative linear regression models incorporating body surface area (BSA)-indexed structural measurements and selected functional indices were evaluated. The aim was to account for model uncertainty by comparing competing models using small sample-corrected information criteria and taking the model average. To enhance transparency and interpretability, these analyses were complemented with SHAP to determine the relative contribution of each predictor within the modelling framework. In this context, the hypothesis of our study was defined as ‘ECHO parameters evaluated in conjunction with body composition are related to aerobic performance.’

## Materials & Methods

### Participants

The study participants were male university students actively involved in sports who were enrolled in a sports science faculty. According to the guidelines of the European Society of Cardiology, individuals who exercise for more than 150 min per week are considered active athletes (Visseren et al., 2021). In this context, university students who exercised for at least 150 min per week were included in the study. Participants completed a medical questionnaire to document any cardiovascular symptoms, family history of acute cardiac death, and other cardiovascular history. Accordingly, participants with thyroid disorders, hypertension, any arrhythmias, any hospitalization due to the cardiovascular disease in the previous year, and heart valve disease were excluded from the study. An *a priori* sample size estimation was performed using G*Power (University of Düsseldorf, Düsseldorf, Germany) for a multiple linear regression model (fixed model, *R^2^* deviation from zero). Assuming a medium-to-large effect size (*f^2^* = 0.35), *α* = 0.05, statistical power (1−*β*) = 0.80, and up to three predictors (consistent with our parsimonious modeling strategy), the required minimum sample size was *n* = 23. *Post-hoc* power analysis was not reported because it is controversial and adds limited interpretive value; instead, overfitting was mitigated by restricting model complexity, Akaike Information Criterion (AICc)-based model comparison/model averaging, and leave-one-out cross-validation (LOOCV).

All participants were informed about the research and its purpose and expected contribution to the literature. Informed consent was obtained from all subjects involved in the study. Written informed consent has been obtained from the participants to publish this paper. All processes in the research were carried out in accordance with the principles set out in the Helsinki Declaration. The necessary permissions for the research were granted by the Band ırma Onyedi Eylül University Health Sciences Non-Interventional Research Ethics Committee with decision number 225/77 (Meeting Date: 14.04.2025).

### Study design

In the present study, the cross-sectional research method was used among the experimental research methods. Therefore, ECHO analyses of all participants were performed within the time frame determined by an expert cardiologist (Kesmodel, 2018). First, demographic information was collected from participants. Then, participants’ resting heart rates (HR), resting peripheral capillary oxygen saturation (SpO_2_), systolic blood pressure (SBP), and diastolic blood pressure (DBP) were measured. Subsequently, all participants underwent ECHO measurements. Following the ECHO measurements, the adaptation phase IFT test was administered. One week later, the IFT test was conducted, and the data were recorded. It was assumed that all participants had slept for 8 h prior to the measurements and were instructed not to consume any food or drink other than water for at least 2 h before the measurements. Participants were informed not to consume products such as alcohol and caffeine that could affect their performance prior to the tests. Participants were asked not to engage in high-intensity exercise for at least 48 h prior to the tests.

### Data collection tools

#### Echocardiographic measurements

All ECHO analyses were performed using a Vivid T8 (GE Healthcare, Milwaukee, Wisconsin, USA) device equipped with a phased array transducer operating in the 2.5–3.5 MHz frequency range. After a 10 minute rest period following admission to the ECHO analysis area, participants underwent analysis by a specialist cardiologist (Lang et al., 2006). Measurements were performed in accordance with the recommendations of the American Society of Echocardiography (ASE), using parasternal long-axis, short-axis, apical two-chamber, and four-chamber views (Rudski et al., 2010). To minimise respiratory-induced variation, each measurement was obtained by averaging three consecutive cardiac cycles at the end of expiration (Chan et al., 2021).

The left ventricular end-systolic diameter (LVSD) and end-diastolic diameter (LVDD), interventricular septum (IVS), and posterior wall (PW) were measured using M-mode in the parasternal long-axis view. Left ventricular end-systolic volume (LVSV) and end-diastolic volume (LVDV) were calculated utilising the modified two-chamber Simpson’s method from apical four- and two-chamber views (Ujino et al., 2006). Additionally, stroke volume (SV) was defined as the difference between LVDV and LVSV. Aortic systolic diameter (ADs) and diastolic (ADd) diameters were measured at the sinotubular junction level in the parasternal long-axis view. The maximum left atrial diameter (LA_max) was recorded at the end of ventricular systole in the apical four-chamber view. Aortic strain (AS) and aortic distensibility (AD) were utilised as aortic elasticity parameters. The following formulas were used to calculate these parameters:

\begin{eqnarray*}AS(\%)= \frac{(ADs-ADd)}{ADd} \times 100 \end{eqnarray*}

\begin{eqnarray*}AD \left( 10-6.cm-2.dyn-1 \right) =2\times \frac{AS}{(SBP-DBP)} . \end{eqnarray*}

Systolic myocardial velocity (Sm), early diastolic myocardial velocity (Em), and late diastolic myocardial velocity (Am) were recorded, and the E/Am ratio was calculated. Isovolumic contraction time (ICRT) and isovolumic relaxation time (IVRT) were determined from Tissue Doppler Imaging (TDI) derived velocity curves. Tricuspid annulus plane systolic excursion (TAPSE) was measured in the apical four-chamber view using M-mode.

Mitral valve inflow was obtained using the pulsatile Doppler method with a sampling volume placed at the level of the mitral valve leaflets in the apical four-chamber view. Early diastolic filling velocity (E) and late diastolic filling velocity (A) were recorded, and the E/A ratio was calculated. In addition, diastolic function indices such as E’/Em were calculated. All measurements were performed by an experienced cardiologist blinded to the participants’ performance test results, and interobserver and intraobserver variation coefficients of <5% were determined for all main parameters in a randomly selected subgroup.

#### Systolic and diastolic blood pressure

SBP and DBP measurements were taken while participants were at rest, in a seated position, and after a minimum rest period of 5 min. Measurements were taken using an automatic oscillometric digital blood pressure monitor (Erka, Ernst Kraemer Co., Bad Tölz, Germany) that applied pressure to the arm using a cuff. Measurements were taken for each participant in the morning at standard room temperature (22–24 °C). Two consecutive measurements were taken from each individual, and a third measurement was taken when the difference between the measurements was greater than five mmHg. Average values were used in the analyses (Johnson et al., 1999).

#### Peripheral oxygen saturation measurement

SpO_2_ measurements were performed using a Philips brand (Philips N.V., Amsterdam, Netherlands) finger pulse oximeter. Measurements were taken while participants were in a comfortable sitting position and had rested for at least 5 min prior to measurement. The device was placed on the index finger of the dominant hand, and the stable value displayed on the screen was recorded. Measurements were repeated both during rest and within the first 30 s following exercise.

#### Heart rate measurements

HR measurements were performed using a Polar H9 chest-type heart rate sensor (Polar Electro Oy, Kempele, Finland). Prior to measurement, the sensor was placed around the chest so that it was in direct contact with the skin and activated according to the manufacturer’s instructions. Heart rate data were recorded at three different time points: (1) at rest, in a sitting position at least 5 min before measurement; (2) during exercise, in the middle of the load; and (3) during the recovery period within the first 30 s after exercise. All measurements were performed under the same environmental conditions and in accordance with the standard protocol (Cheatham, Kolber & Ernst, 2015).

#### 30-15 intermittent fitness test

The 30-15 IFT protocol was applied to assess participants’ aerobic power and high-intensity interval running capacity. The test was conducted on a flat running track 40 m in length. Cones were placed every 5 m along the track, and a 3 m turning area was created at both ends. At the start of the test, participants ran forward and backward for 30 s in sync with an audible beep system; a 15 s passive recovery period followed each running period. The running speed was initially set at 8 km/h and increased by 0.5 km/h every 30 s running period. Participants were required to maintain the running speed and timing in accordance with the audio signal. The test was terminated if the participant failed to reach the audio signal on time twice in a row, voluntarily withdrew from the test, or was stopped by the trainer for medical or physical reasons. The maximum speed reached by the participant in the test (IFT) was recorded as the speed of the last completed stage (Buchheit, 2008). This value was used to determine aerobic capacity and adjust training load intensities. All tests were performed on a soccer field under similar environmental conditions (20–22 °C, 50–60% relative humidity) and when participants were rested, well fed, and hydrated. The 30-15 IFT original test sound file was used as the audio signal system. Participants’ HR results were also recorded during the test along with IFT.

### Statistical analyses

All statistical analyses were performed using R software (R Foundation for Statistical Computing, Vienna, Austria). Within this scope, the tidyverse, MuMIn, broom, performance, sandwich, lmtest, boot and ggplot2 packages were used for data cleaning, model selection/model averaging, robust inference, cross-validation and visualisation. Continuous variables were reported as mean ± standard deviation and minimum–maximum. To reduce scale differences related to body size in echocardiographic structural measurements, variables indexed by BSA were prioritised and these indexed parameters were used in the analyses. The primary dependent variable of the study was 30–15 IFT performance.

To examine the echocardiographic predictors associated with IFT in a manner that minimises the risk of overfitting under small sample conditions, clinically interpretable parsimonious linear regression models were constructed. The candidate model set consisted of models formed from a predefined variable pool, each containing a limited number of predictors (parsimonious). The small sample adjusted AICc was used to compare the models; ΔAICc (difference from the best model) and Akaike weight (w) were calculated for each model to quantitatively express relative model support. To reflect model uncertainty, a model averaging approach was adopted using AICc weights instead of relying on a single ‘best model’.

The importance of variables was planned to be reported using two complementary indicators: (i) Σw, the AICc-weighted model average importance measure, summarises a variable’s frequency of inclusion in supported models and its relative contribution; (ii) SHAP were used for the explainability of the contribution to the model output, and the contribution magnitude of the variables was reported using the weighted mean(—SHAP—). To summarise the direction of the variables’ relationship, the regression coefficients obtained from the candidate models were combined with Akaike weights to calculate the weighted average coefficient (β weighted avg). Thus, the results were reported jointly based on both model support and explainability measures.

To assess model generalisability under small sample conditions, LOOCV was applied for each model, and root mean squared error (RMSE) was reported as the performance metric. To control for the possibility of multicollinearity, the variance inflation factor (VIF) was calculated for the top-ranked models, and a VIF < 5 threshold was used in the collinearity assessment. A two-tailed statistical approach was adopted in all analyses.

## Results

Table 1 shows the descriptive statistics of the participants. The mean age of the participants was 20.47 ± 1.53 years, height was 175.56 ± 9.29 cm, body weight was 73.21 ± 15.91 kg, and body composition (BMI) was 23.55 ± 3.73 kg/m^2^. The mean resting systolic blood pressure was 123.82 ± 11.53 mmHg, and the mean diastolic blood pressure was 72.82 ± 11.18 mmHg. Resting heart rate was measured as 78.17 ± 9.74 beats/min, and resting SpO_2_ value was measured as 98.17 ± 0.93%. After IFT, the average heart rate increased to 180.95 ± 15.23 beats/min, while the SpO_2_ value was recorded as 95.69 ± 2.11%. In relation to aerobic function and echocardiographic properties, the average performance in the IFT test was 17.35 ± 1.85, reflecting VO_2_max estimation of 46.07 ± 4.85 ml/kg/min. The indexed parameters for left ventricular structural parameters showed that the average value for the left ventricular end-diastolic diameter (LVEDD) index was 25.25 ± 3.03, with the left ventricular end-systolic diameter (LVESD) index being 16.14 ± 2.30, and the septal and posterior wall thickness indices demonstrated values of 5.43 ± 0.80 and 5.18 ± 0.77, respectively. The indexed measures for the ventricles reflected volumes of 56.30 ± 10.25 for the LVEDV index and 16.14 ± 2.30 for the LVESV index, translating to the indexed stroke volume of 37.26 ± 9.31. The left ventricular function during the systolic phase remained intact, with the diastolic velocity parameter values obtained by Doppler techniques included a mean E and A of 0.91 ± 0.10 m/s and 0.63 ± 0.12 m/s, respectively, yielding an E/A ratio of 1.47 ± 0.22. Velocity data for E′, A′, and S′, obtained by Tissue Doppler echocardiography, indicated a mean E′ of 13.91 ± 2.57 cm/s, A′ of 8.13 ± 1.63 cm/s, and S′ of 9.57 ± 1.78 cm/s, and an E′/A′ ratio of 1.77 ± 0.46. The mean IVRT and ICRT duration for the isovolumetric relaxation and contraction periods, respectively, were 109.22 ± 28.17 ms for IVRT and 83.56 ± 12 for ICRT. Aortic and left atrial variables showed an average aortic systolic diameter of 28.00 ± 3.32 mm and an average aortic diastolic diameter of 23.00 ± 3.16 mm. On measurement of aortic elasticity, the average aortic strain was 22.21 ± 7.26 and aortic distensibility of 22.39 ± 9.93. The mean maximum diameter of the left atrial dimension was 32.48 ± 3.72 mm.

**Table 1:** Baseline information of participants.

**Parameters**	**Mean**	**S.D.**	**Minimum**	**Maximum**
Age (year)	20.47	1.53	18.0	25.0
Height (cm)	175.56	9.29	160.0	194.0
Weight (kg)	73.21	15.91	52.0	120.0
BMI (kg/m^2^)	23.55	3.73	17.92	37.45
SBP (mmHg)	123.82	11.53	100.0	150.0
DBP (mmHg)	72.82	11.18	50.0	100.0
HR_rest_ (pulse)	78.17	9.74	52.0	97.0
SpO_2rest_	98.17	0.93	96.0	99.0
HR_post_ (pulse)	180.95	15.23	137.0	198.0
SpO_2post_	95.69	2.11	92.0	99.0
IFT	17.35	1.85	11.5	20
VO2max (ml kg^−^^1^ min^−^^1^)	46.07	4.85	32.3	52.8
LVEDD index	25.25	3.03	18.25	30.52
LVESD index	16.14	2.3	11.07	19.34
IVS index	5.43	0.8	3.17	6.92
PW index	5.18	0.77	3.17	6.32
LVEDV index	56.3	10.25	38.11	77.08
LVESV index	16.14	2.3	11.07	19.34
SV index	37.26	9.31	25.67	68.23
LVEF (%)	65.78	5.78	55	75
E (m/s)	0.91	0.1	0.62	1.1
A (m/s)	0.63	0.12	0.45	0.92
E’ (cm/s)	13.91	2.57	9	19
A’ (cm/s)	8.13	1.63	5	11
S’ (cm/s)	9.57	1.78	7	13
IVRT (ms)	109.22	28.17	59	171
ICRT (ms)	83.56	12.01	61	100
E/A ratio	1.47	0.22	0.98	2
E’/A’ ratio	1.77	0.46	1.09	2.83
TAPSE (mm)	25.18	5.21	18	43
Aortic systole (mm)	28	3.32	22	35
Aortic diastole (mm)	23	3.16	19	29
Aortic strain	22.21	7.26	9.09	36.84
Aortic distensibility	22.39	9.93	7.27	44.21
LA max (mm)	32.48	3.72	26	40

**Notes.**

BMI Body Mass Index DBP Diastolic Blood Pressure HR Heart Rate SpO_2_ Peripheral Capillary Oxygen Saturation LVEF Left Ventricular Ejection Fraction SBP Systolic Blood Pressure

**Figure 1:**
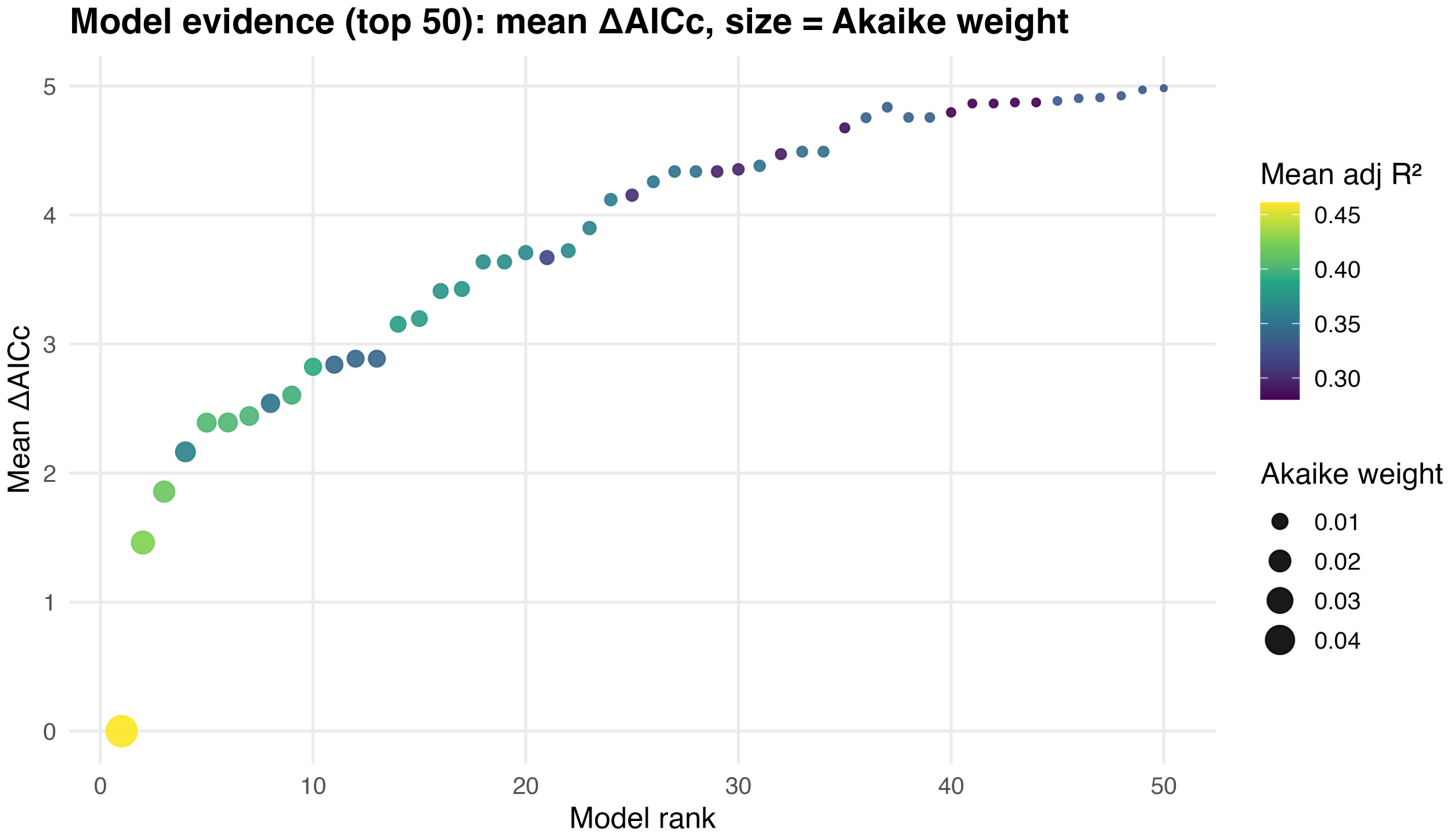
Model evidence plot based on AICc (cumulative Akaike weights) across candidate models.

Table 2 shows the top 10 parsimonious linear regression models with the highest support out of the candidate models assessed to describe the performance of IFT, their performance, and comparison with each other. The best model included the variables BMI + PW_I + aortic strain (AORT_STR), explaining about 46% of the IFT performance, with avg. adj. *R*^2^ = 0.461; LOOCV RMSE = 1.811 for the same model. This is followed by the model with the subsequent best level of support, BMI + PW_I + E_A, with ΔAICc = 1.462; weight = 0.0229; avg. adj. *R*^2^ = 0.426; LOOCCV RMSE = 1.834. In the third best model, IVS_I replaced the variable PW_I, with the model BMI + IVS_I + AORT_STR, ΔAICc = 1.856; avg. adj. *R*^2^ = 0.416; LOOCV RMSE = 1.896. In the top models with only two variables, the best model included the variables PW_I + E_A, ΔAICc = 2.165; avg. adj. *R*^2^ = 0.360, with one of the lowest error levels, namely, LOOCV RMSE = 1.615; the model with the remaining two variables, namely, PW_I + AORT_STR, also has low error level, namely, LOOCV RMSE = 1.628. All the top 10 models included the variables for BMI, the wall thickness indices (PW_I or IVS_I), diastole indices (A or E_A), and/or the aorta elasticity indicator (AORT_STR) in various combinations; however, the low weights, with the large spread, suggest the presence of model uncertainty. Figure 1 visually supports the comparison of the top 10 parsimonious models presented in Table 2 and demonstrates a clear model uncertainty among the candidate models. The fact that multiple models receive similar levels of support within a low ΔAICc range and that Akaike weights do not concentrate on a single model indicates that there are multiple plausible models rather than a single best model in explaining IFT. Therefore, in interpreting the findings, the distribution of model support weights should be considered alongside the model ranking.

**Table 2:** Results of the GAM model for IFT estimation using routine structural ECHO parameters.

Rank	Predictors	Mean ΔAICc	Mean Akaike weight	Mean adj R^2^	LOOCV RMSE
1	BMI + PW_I + AORT_STR	0	0.0476	0.461	1.811
2	BMI + PW_I + E/A	1.462	0.0229	0.426	1.834
3	BMI + IVS_I + AORT_STR	1.856	0.0188	0.416	1.896
4	PW_I + E/A	2.165	0.0161	0.36	1.615
5	BMI + IVS_I + LVEF	2.391	0.0144	0.402	1.868
6	BMI + IVS_I + A	2.392	0.0144	0.402	1.985
7	BMI + PW_I + A	2.442	0.014	0.401	1.977
8	PW_I + AORT_STR	2.541	0.0134	0.35	1.628
9	BMI + LVEF + A	2.604	0.013	0.396	1.944
10	BMI + PW_I + LVEF	2.824	0.0116	0.391	1.9

**Notes.**

ADd Aortic diameter diastole ADs Aortic diameter systole IVS Interventricular septal thickness LA_max Left Atrial Maximum Diameter LVDD Left ventricular end-diastolic diameter LVEF Left Ventricular Ejection Fraction LVSD Left ventricular end-systolic diameter LVDV Left ventricular end-diastolic volume LVSV Left ventricular end-systolic volume PW Posterior wall thickness SV Stroke Volume

**Figure 2:**
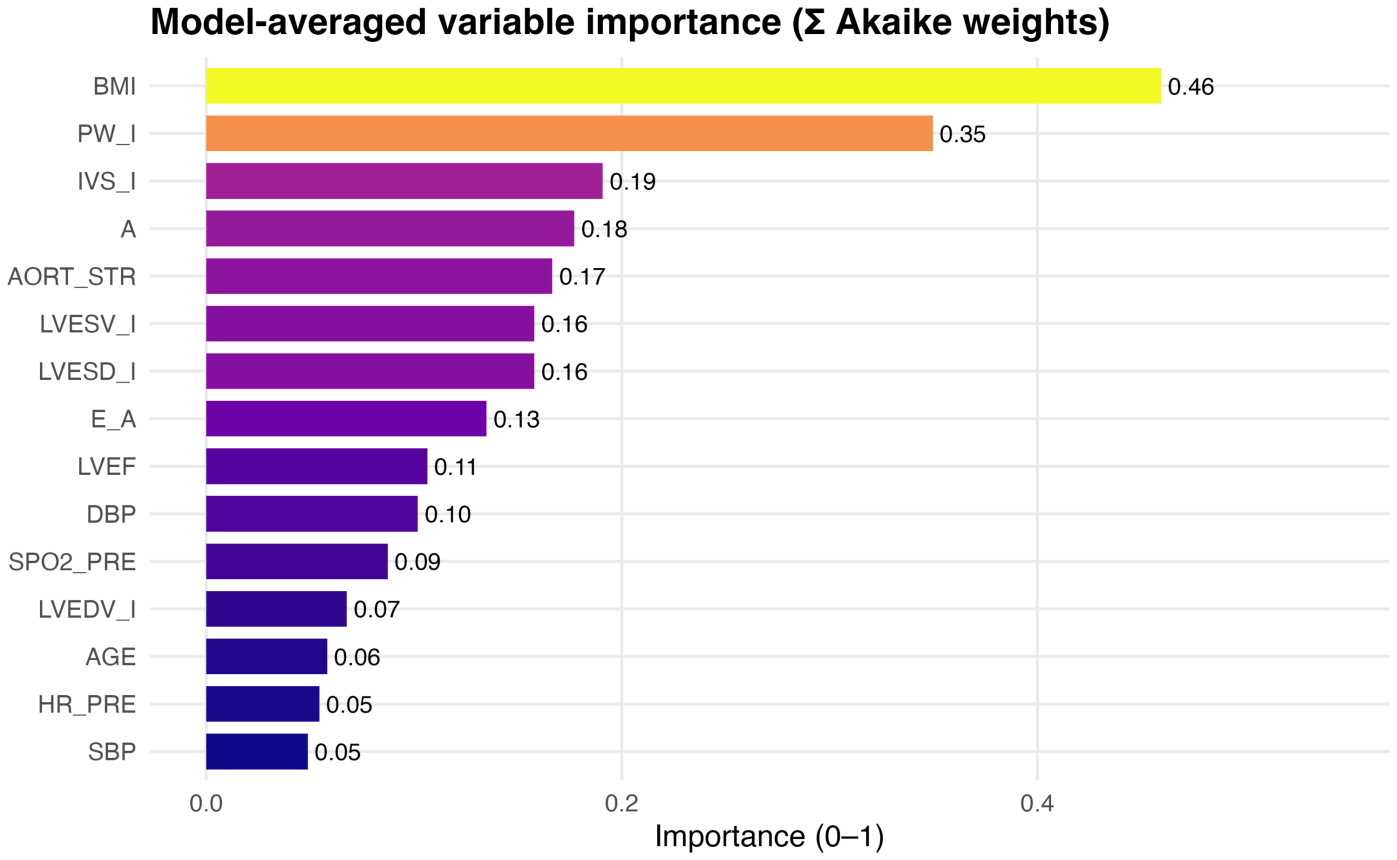
Variable importance based on model-averaged Akaike weights (Σ w) across candidate models predicting 30–15 IFT performance.

**Figure 3:**
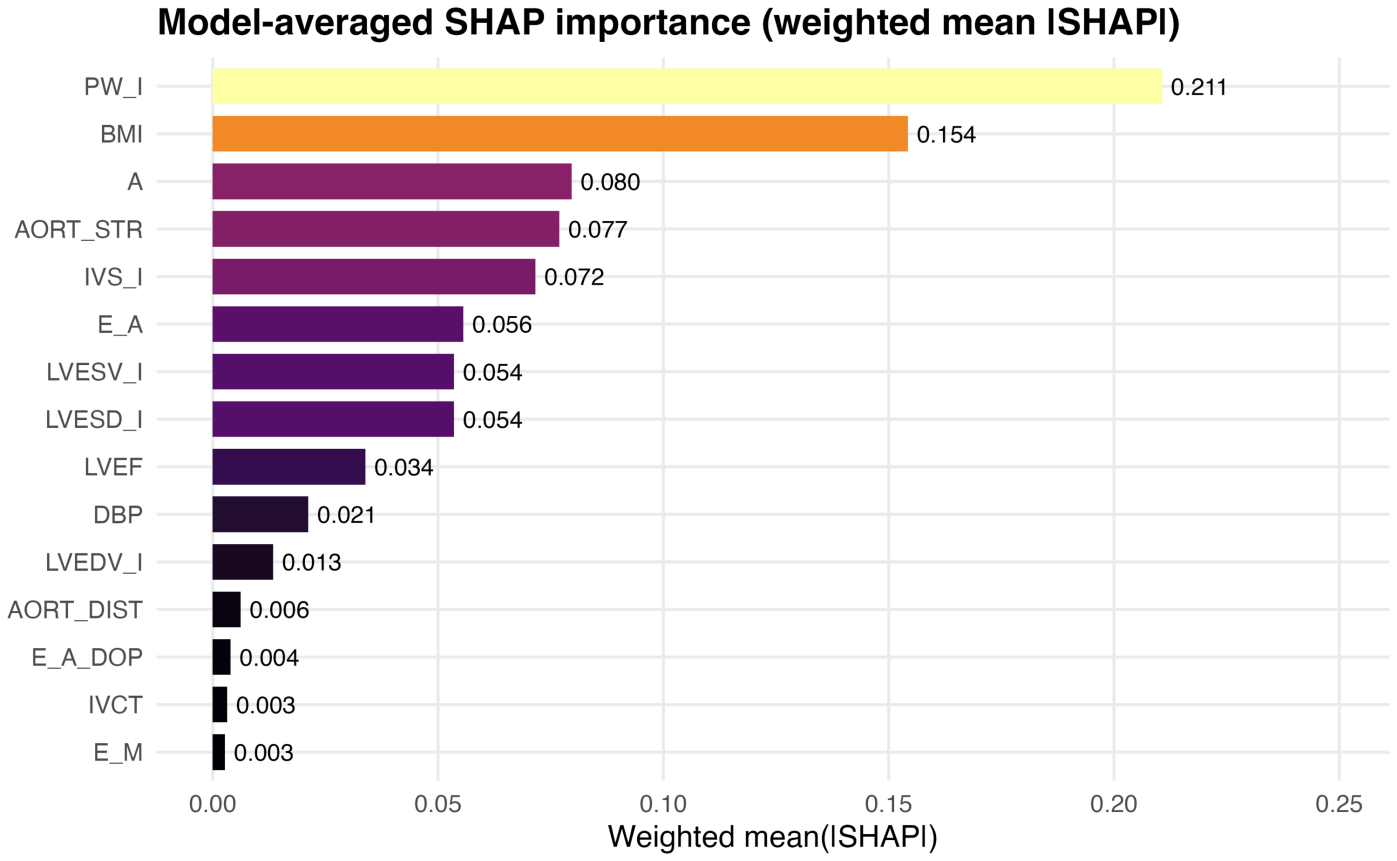
Weighted SHAP feature importance across candidate models predicting 30–15 IFT performance (weighted mean absolute SHAP values).

Table 3 summarises the consistency level of variables, model-average importance, and direction of relationship in candidate models evaluated to explain IFT performance. The model-average importance measure Σw indicates how frequently and how strongly the relevant variable is present in well-supported models; according to this measure, BMI (Σw = 0.46) and posterior wall thickness indexed by BSA (PW_I; Σw = 0.35) emerged as the most consistent predictors associated with IFT. These were followed by IVS_I (Σw = 0.191), the A wave associated with diastolic filling (Σw = 0.177), and AORT_STR (Σw = 0.166), reflecting aortic elasticity. While indexed systolic size/volume measures such as LVESD_I/LVESV_I showed a lower but consistent contribution (Fig. 2). This set of findings suggests that, in relation to IFT, BMI and indexed structural cardiac remodelling indicators (wall thickness indices) in particular provided a more consistent signal, while some diastolic flow and aortic elasticity indicators contributed to a lesser extent. The contribution of the same variables to the model is also supported by the weighted mean (—SHAP—) values calculated using the model-average approach; this metric indicates how much the variable ‘pulls’ the estimated IFT value on average (the magnitude of the contribution). In the SHAP results, PW_I (0.211) and BMI (0.154) stood out as the variables with the highest contribution size, followed by A (0.080), AORT_STR (0.077) and IVS_I (0.072); this ranking indicates that variable importance is not specific to a single model and that a certain core group of variables stands out even under model uncertainty (Fig. 3). From a directional analysis perspective, the negative weighted coefficient of BMI (*β* =−0.068) indicates a tendency for IFT to decrease as BMI increases, while the positive coefficients of indexed wall thickness measures such as PW_I (*β* = 0.348) and IVS_I (*β* = 0.124) suggest that they may be associated with higher IFT. Conversely, the negative direction of the E/A ratio (*β* =−0.333) can be interpreted as a signal indicating that differences in the diastolic filling pattern may be inversely related to IFT. However, due to the limited sample size and the distribution of model weights, these findings should be interpreted as relationship patterns that consistently emerge in a small sample rather than causal inferences.

**Table 3:** Results of the GAM model for IFT estimation using LV systolic, diastolic, and tissue doppler parameters.

Predictor	Σw (Model-averaged importance)	Weighted mean (—SHAP—)	*β* (weighted avg)	Direction
BMI	0.46	0.154	−0.068	Negative
PW_I	0.35	0.211	0.348	Positive
IVS_I	0.191	0.072	0.124	Positive
A-wave	0.177	0.08	0.849	Positive
AORT_STR	0.166	0.077	0.013	Positive
LVESD_I	0.158	0.054	0.03	Positive
LVESV_I	0.158	0.054	0.03	Positive
E/A	0.135	0.056	−0.333	Negative
LVEF	0.106	0.034	−0.007	Negative
DBP	0.102	0.021	0	Positive
SPO2_PRE	0.087	0.003	0.003	Positive
LVEDV_I	0.068	0.013	0.002	Positive

**Notes.**

A Late diastolic flow parameter Am Peak late diastolic AD Aortic Distensibility AS Aortic Strain Em Peak early diastolic E Early diastolic flow velocity ICRT Isovolumic contraction time IVRT Isovolumic relaxation time Sm Peak early systolic TAPSE Tricuspid Annular Plane Systolic Excursion BSA Body surface area BMI Body Mass Index PW_I BSA–indexed left ventricular posterior wall thickness IVS_I BSA–indexed interventricular septal thickness A Late diastolic transmitral inflow velocity AORT_STR Aortic strain LVESD_I BSA–indexed left ventricular end-systolic diameter LVESV_I BSA–indexed left ventricular end-systolic volume E_A Transmitral E/A ratio LVEF Left ventricular ejection fraction DBP Diastolic blood pressure SPO2_PRE Resting peripheral capillary oxygen saturation (SpO_2_) LVEDV_I BSA–indexed left ventricular end-diastolic volume

## Discussion

The findings of this study indicate that aerobic performance (IFT) in amateur/active athletes is too multidimensional to be explained by a single EKO measure; however, when certain structural and functional indicators are evaluated together, they provide a more consistent signal. The AICc-based model comparison and model average approach revealed that the models with the highest support for explaining IFT performance mostly included indices of left ventricular wall thickness indexed by BMI and BSA (particularly PW_I and IVS_I), which reflect body composition, and secondarily diastolic filling indices (A wave, E/A) with aortic elasticity (AORT_STR) and diastolic filling indicators (A wave, E/A). The prominence of variable importance, both in terms of model-average Σw and SHAP contribution magnitudes, supports that the findings are not specific to a single model and that a certain ‘core’ group of variables may be associated with performance even under model uncertainty. In this regard, we believe that the study offers a reliable approach to evaluating ECHO-performance relationships within a conservative and interpretable framework, which may mitigate the risk of overfitting that can arise from complex/highly flexible models under small sample conditions.

In this study, EKO indicators explaining the 30–15 IFT performance were evaluated using an information-criterion-based parsimonious modelling approach that minimises the risk of overfitting under small sample conditions. It is classically emphasised that AICc offers a more appropriate correction than AIC in small samples and that inference based on a model set is more realistic than a ‘single best model’; uncertainty can be quantitatively transferred with Akaike weights (wi) (Hurvich & Tsai, 1989; Burnham & Anderson, 2004). Within this framework, the fact that model evidence is not concentrated in a single model is to be expected in multi-component performance outputs such as IFT; because aerobic field performance can be shaped simultaneously by a combination of body composition, cardiac structure-function, and vascular characteristics. The ‘athlete’s heart’ literature also clearly demonstrates that adaptations in athletes cannot be reduced to a single parameter, and that the echocardiographic phenotype is multidimensional (Persch & Steinacker, 2020; Flanagan et al., 2023).

In this study, the prominence of BMI in the model mean and SHAP results supports the decisive role of body mass/composition in performance in field tests such as the 30–15 IFT, which are running-based and involve repeated acceleration–deceleration. It has previously been demonstrated that 30–15 IFT performance is influenced by body mass and that the effect of BMI must be considered when interpreting results (Darrall-Jones et al., 2016). Similarly, field studies reporting significant correlations between body composition and BMI with 30–15 IFT performance emphasise that this test reflects not only cardiorespiratory capacity but also mechanical loading and movement economy (Silva et al., 2022). In this context, the relationship between BMI and IFT is not a noise factor independent of ‘cardiac capacity’; rather, it should be interpreted as a genuine performance component that influences running economy, the cost of changing direction, and relative loading. From a physiological mechanism perspective, as BMI (and the accompanying fat mass/fat-free mass distribution) increases, the mass carried during running increases, and this situation may raise the energy cost per unit distance; consequently, maintaining speed increases may become difficult, particularly in intermittent protocols. Studies examining the relationship between body mass and anthropometry with running economy/energy cost have reported that increases in mass may have significant effects on running cost and that performance-related anthropometric profiles may vary by sport (Maldonado, Mujika & Padilla, 2002). On the other hand, the prominence of BMI does not imply that EKO parameters are insignificant; rather, it suggests that BMI must be included in the model as a contextual variable (the mechanical/anthropometric component of performance) in order to correctly interpret the EKO–performance relationship. Indeed, in field tests such as the 30–15 IFT, performance is a combination of elements such as sprint repetition, change of direction and running economy, in addition to cardiac-circulatory capacity; the effect of body mass and composition can be particularly pronounced in this combination.

When we interpret our findings in the context of FT from a physiological perspective, the negative correlation between BMI and performance, coupled with the positive correlation observed for BSA-indexed wall thicknesses (PW_I, IVS_I), does not represent a contradiction; rather, it is the expected outcome of simultaneously measuring the ‘mechanical load–cardiac adaptation’ axes. The 30–15 IFT is a test that can suppress performance through running economy and the cost of changing direction as body mass increases, due to its intermittent structure and the requirement for repeated acceleration/deceleration; indeed, it has been demonstrated that body mass significantly influences the interpretation of VIFT/IFT in the 30–15 IFT, and that performance comparisons change meaningfully when mass covariates are added (Stanković et al., 2021). In addition, BMI (and particularly the increased haemodynamic load associated with adiposity) is a factor that can increase wall thicknesses in a ‘pathophysiological’ direction; current data showing the relationship between obesity/BMI and LV hypertrophy, septal and posterior wall thickness support the notion that wall thickness can increase as BMI rises (Cai et al., 2024).

However, precisely for this reason, indexing PW and IVS according to BSA becomes critical: indexing reduces the bias of ‘larger bodies naturally appearing to have thicker walls’ and, after controlling for the performance-reducing mechanical effect of BMI, allows the remaining positive signal in wall thickness to represent the training-related physiological remodelling/contractile reserve dimension. It is widely emphasised in the literature that physiological remodelling in athletes can be shaped through wall thickness and geometry; that this varies according to the sport and type of training; and that in most athletes, it remains within physiological limits (Pluim et al., 2000; Baba Ali et al., 2024). Within this framework, while it is expected that IFT performance will decrease with increasing BMI in terms of ‘carried mass and metabolic cost,’ when BMI/BSA scale effects are separated, PW_I and IVS_I stand out positively, indicating that individuals with more favourable cardiac structural adaptation at the same BMI level may have an advantage in intermittent aerobic performance; that is, while BMI lowers performance, indexed wall thickness may support systolic pressure development and haemodynamic endurance during repeated efforts in some athletes through ‘adaptive remodelling’ (Abibillaev et al., 2024). The 30-15 IFT rapidly increases heart rate and shortens diastole due to short intervals of increasing speed and recovery periods; to maintain performance under these conditions, sufficient filling (preload) + effective emptying + low/compatible afterload balance must be maintained in each repetition/phase (Stanković et al., 2021). Therefore, the lower but recurrent A wave and E/A ratio observed in the model set suggest that during IFT, the shortened diastole may become more dependent on atrial contribution (A-wave) rather than just early passive filling (E) for ventricular filling; particularly as load and heart rate increase, the convergence/fusion of the E and A waves may lead to filling dynamics becoming more pronounced in performance (Hoffmann et al., 2021; Ferrara et al., 2025). Similarly, the AORT_STR signal is consistent with the mechanism whereby a more elastic proximal aorta could improve ventricular–vascular matching during afterload fluctuations in each phase of intermittent running, thereby making systolic ejection ‘more economical’: when aortic elasticity is high, pulsatile load decreases, effective arterial load decreases, and a more stable stroke volume/blood flow can be maintained with the same cardiac effort; this contributes to maintaining performance during repeated high-intensity running phases (Pugliese et al., 2022; Wong et al., 2025). In contrast, the more limited appearance of systolic measures such as left ventricular ejection fraction (LVEF) and LVESD_I/LVESV_I can be explained by the fact that EF mostly remains within a narrow range in the young-active sample and may be ‘less sensitive’ in distinguishing performance differences; therefore, current sports cardiology and echocardiography literature emphasises that EF alone may not be sufficient, particularly in capturing subtle/functional differences, and that strain-based measurements may be more sensitive (Mihos et al., 2025).

This study should be evaluated within certain methodological and practical limitations. Firstly, the study sample was limited to only 23 active male athletes. This prevented the identification of potential differences between different sports, training histories, or, in particular, gender groups. Research conducted with a larger and more heterogeneous sample would increase the generalisability of the findings. Secondly, although the 30–15 IFT used in this study is a reliable method for assessing aerobic capacity in the field, the lack of direct measurement of gas exchange parameters (*e.g.*, VO_2_max) led to the interpretation of cardiorespiratory capacity based solely on indirect indicators. In future studies, the use of direct gas analysis methods alongside IFT may contribute to strengthening the results. Thirdly, in this study, ECHO parameters were obtained only under resting conditions. The exclusion of acute measurements during or after exercise may lead to the disregard of more dynamic information about the heart’s actual performance capacity. Finally, the cross-sectional design of the study does not permit the establishment of definitive causal relationships. Longitudinal designs or intervention studies are required to assess the causal relationship between cardiac morphology and aerobic performance. This research was conducted on young male amateur athletes. In this context, studies comparing different genders and different populations are also needed in order to generalise the research findings.

## Conclusions

The findings of this study indicate that 30–15 IFT performance cannot be explained by a single echocardiographic measure; rather, it is influenced by parameters reflecting aortic elasticity (AORT_STR) and filling dynamics (A and E/A), particularly those indexed to body size/composition (BMI) and BSA, such as left ventricular wall thickness indices, (PW_I and IVS_I), parameters reflecting aortic elasticity (AORT_STR), and those representing filling dynamics (A and E/A). Parsimonious modelling, generalisability control with LOOCV, and the explainability approach with SHAP have enabled the transparent presentation of dominant signals supporting IFT performance by reducing overfitting under small sample conditions. These results suggest that in performance evaluation, ECHO may serve not only as a clinical monitoring tool but also as a framework to help define the performance-related cardiovascular profile in athletes. From a practical standpoint, these findings suggest that the combined application of planned endurance training aimed at improving body composition and intermittent high-intensity interval training may contribute to IFT performance by supporting echocardiographic indicators related to ventricular filling dynamics and vascular elasticity. However, as this study is cross-sectional, these suggestions should be interpreted as correlational rather than causal and should be confirmed by longitudinal/experimental studies.

##  Supplemental Information

10.7717/peerj.21263/supp-1 Supplemental Information 1 Raw data

## Abbreviations

ECHO: echocardiographic
IFT: intermittent fitness test
SHAP: SHapley Additive exPlanations
LV: left ventricular
IVRT: isovolumic relaxation time
ICRT: isovolumic contraction time
E: early diastolic filling velocity
A: late diastolic filling velocity
Em: early diastolic myocardial velocity
Am: late diastolic myocardial velocity
Sm: systolic myocardial velocity
TAPSE: tricuspid annular plane systolic excursion
LVSD: left ventricular end-systolic diameter
LVDD: left ventricular end-diastolic diameter
IVS: interventricular septum thickness
PW: posterior wall thickness
LVDV: left ventricular end-diastolic volume
LVSV: left ventricular end-systolic volume
SV: stroke volume
ADs: aortic end-systolic diameter
ADd: aortic end-diastolic diameter
LA_max: maximum left atrial diameter.

## Additional Information and Declarations

### Data Availability

The following information was supplied regarding data availability: The raw data are available in the Supplemental File.
